# Patient gender preferences in neurosurgical care: A cross-sectional study with operational modelling

**DOI:** 10.1016/j.bas.2026.106035

**Published:** 2026-04-09

**Authors:** Aaron Lawson McLean, Anne Neumeister, Julian Kahr, Anna C. Lawson McLean, Nicole Demme-Krüger, Rebecca Liebscher, Christian Senft

**Affiliations:** Department of Neurosurgery, Jena University Hospital, Friedrich Schiller University Jena, Jena, Germany

**Keywords:** Neurosurgery, Patient preferences, Physician gender, Patient-centred care, Health services research

## Abstract

**Introduction:**

Physician gender may influence patient comfort and communication dynamics in clinical encounters. However, empirical evidence on physician-gender preferences in procedural, high-technology specialties such as neurosurgery remains limited, particularly in outpatient care.

**Research question:**

What is the prevalence of physician-gender preferences among neurosurgical outpatients across clinical scenarios, which factors are associated, and what are the operational implications?

**Material and methods:**

We conducted a cross-sectional survey of 324 adult neurosurgical outpatients at a German university hospital. Respondents reported physician-gender preferences across five scenarios: consultation, examination, minor intervention, major operation, and disclosure of bad news. Multivariable logistic regression assessed demographic, clinical, and affective associations. Monte Carlo simulations estimated preference fulfilment under varying physician gender ratios.

**Results:**

Between 32.7% and 48.1% of respondents reported a physician-gender preference. Directional preferences were evenly split between female and male physicians. Demographic factors, education, and disease category showed no consistent associations. Symptom-related worry increased preference expression for minor interventions (OR 1.65, 95% CI 1.03–2.66) but reduced it for major operations (OR 0.58, 95% CI 0.36–0.94). Male patients were less likely to express preferences in operative settings (OR 0.55, 95% CI 0.34–0.89). Simulations showed that altering workforce gender ratios changed fulfilment rates by less than two percentage points.

**Discussion and conclusion:**

Physician-gender preferences are common but balanced and weakly associated with patient characteristics. Emotional context rather than physician gender drives preference expression. Adjusting workforce composition offers minimal benefit; attention to situational anxiety and high-quality communication is more impactful for patient-centred neurosurgical care.

## Highlights

• One-third to one-half of neurosurgical outpatients report physician-gender preferences.
• Direction of preference is balanced between female and male physicians.
• Preferences are driven by situational anxiety, not patient demographics.
• Changing staff gender ratios minimally affects preference fulfilment rates.

## Graphical abstract

**Figure:**
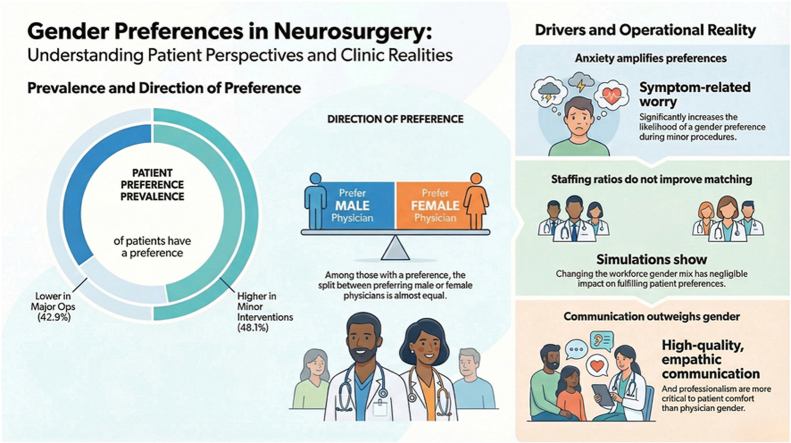

## 1 Introduction

Patient-centred neurosurgical care inherently extends beyond technical operative expertise: it demands effective communication, careful physical examination, and management of high-stakes decision-making that often involves substantial vulnerability. In neurosurgery, patients may face neurological deficits, the prospect of major surgery, or fear of tumour progression, and these scenarios can evoke heightened sensitivity regarding physician–patient interaction. In other clinical domains, research indicates that physician gender may play a role in patient comfort and disclosure: for example, a U.S. primary-care study found that both male and female patients often preferred a same-gender provider, with preference more pronounced among men (Fink et al., 2020). Another cross-sectional Brazilian study found that 81.7% of patients reported no physician-gender preference overall, but among those who did the rate was elevated in specialties involving intimate examination (33.4% versus 9.7%) (Dagostini et al., 2022). These findings underline that while most patients might not explicitly choose a physician gender, a meaningful minority do—especially in contexts entailing bodily exposure or emotionally charged communication.

Despite this literature, specialty-specific evidence in procedural or high-technology fields such as neurosurgery remains sparse. A recent mixed-methods study from Taiwan reported that roughly one-third of neurosurgical outpatients preferred a female neurosurgeon, particularly female patients, with privacy, modesty, and communicative empathy cited as key motives (Lee et al., 2024). Such findings likely reflect sociocultural contexts in which gender norms and patient–physician hierarchies remain more pronounced. By contrast, in Germany, where gender equality in medicine is an explicit institutional goal but remains incompletely realised, women account for only about 23% of senior neurosurgical specialists (Weiss et al., 2023). This divergence between cultural expectations of gendered interaction and structural representation within the workforce underscores why physician-gender preference in neurosurgery may manifest differently across settings.

Accordingly, the present study aimed to quantify (1) the prevalence of physician-gender preference across five clinically relevant neurosurgical scenarios (consultation, examination, minor intervention, major operation, and disclosure of bad news), (2) the correlates of both any preference and directional preference (toward female vs male physicians) including demographic, symptom burden, disease category and prior request behaviour, and (3) the operational implications by estimating the appointment-matching capacity required under realistic staffing mixes to honour declared preferences. Through this three-pronged approach we seek not only to document the phenomenon but to provide actionable guidance for neurosurgical outpatient services.

## 2 Methods

### 2.1 Study setting

The study was conducted in the outpatient clinics of a university-affiliated neurosurgical centre in Thuringia, Germany. The institution is a tertiary academic referral centre serving a largely regional catchment in central Germany. Thuringia has a population of approximately 2.1 million (2024 estimate) with a nearly balanced gender distribution (≈49% male, 51% female) and an older age structure than the national average (Eurostat, 2025; Demografieportal des Bundes und der Länder, 2025; Thüringer Landesamt für Statistik, 2023). The region includes moderately dense urban centres (e.g. Erfurt, Jena) and extensive rural areas, encompassing diverse socioeconomic profiles with a strong manufacturing base and expanding academic sector. The patient population attending the outpatient service thus reflects a typical mix of urban and semi-rural German residents. Consecutive adult outpatients were invited to participate anonymously during routine visits. No formal exclusion criteria were applied other than the ability to complete the questionnaire independently, and although approach counts were not recorded to preserve anonymity, approximately 110–120 patients attend the outpatient service per week, providing insight into recruitment flow and representativeness. Physicians in the outpatient clinic rotate regularly, and patients are therefore commonly seen by different physicians across visits rather than by a single, continuously assigned provider. The study protocol was approved by the responsible institutional ethics committee (reference 2023-2910-Bef).

### 2.2 Participants and survey instrument

All consecutive clinic attendees during the two-month recruitment period in the year 2023 were invited to complete a brief, anonymous questionnaire (Supplementary File 1). The instrument assessed, for five clinical scenarios—consultation, examination, minor intervention under local anaesthesia, major operation under general anaesthesia, and disclosure of bad news—whether respondents had any physician-gender preference and, if so, its direction (male or female physician). “Minor intervention” referred to brief, low-risk neurosurgical procedures typically performed under local anaesthesia in the outpatient or short-stay setting, such as diagnostic or therapeutic infiltrations, peripheral nerve decompressions, or biopsies, and was presented as a generic category rather than a specific procedure.

Demographic variables included patient gender, age group, disease category, relationship status, general practitioner (GP) status, education, religion, and immigration-related background. Contextual indicators captured whether the presenting problem limited daily activities, worried the patient, was painful, or was embarrassing. The item “symptom is worrying” was conceptually intended to represent perceived symptom severity and situational anxiety, aligning with affective dimensions relevant to patient comfort and communication. Additional items recorded first-visit status and, for return visits, the gender of the physician at the previous encounter and whether the patient believed the advice might have differed with a physician of another gender.

The questionnaire underwent structured content mapping to the study aims and face validation by three neurosurgeons representing neuro-oncology, spine, and functional subspecialties, together with a senior clinic nurse. Items were evaluated for clarity, redundancy, and workflow compatibility. A pilot test with 15 patients resulted in minor wording revisions. These steps established both face and content validity. The survey was fully anonymous, integrated into the normal clinic workflow, and required no written consent. Because approach counts were intentionally not recorded, response and consent rates could not be calculated.

### 2.3 Outcomes

Two outcomes were defined for each clinical scenario:

(1) Presence of any physician-gender preference (yes/no).
(2) Among those with a preference, direction of preference (female vs male physician).

### 2.4 Statistical analysis

All analyses were conducted in Python 3.11 (Python Software Foundation, Wilmington DE, USA) using pandas, numpy, statsmodels, and matplotlib. For each scenario, a prespecified multivariable logistic regression model was fitted with the following predictors: patient gender, age group, disease category, daily-activity limitation, worry, pain, embarrassment, first-visit status, immigration background, and prior specific-gender request. Categorical variables were dummy-coded with fixed reference levels (female gender, age 20–39 years, disease category “other”). Odds ratios (ORs) with HC3-robust 95% confidence intervals and two-sided Wald p values were reported. The “diverse” gender category was analysed descriptively but excluded from regression modelling because of its very small number (n = 1). Missingness for covariates (0.0–1.3%) was handled by mode imputation.

An expanded model additionally included education, religion, relationship status, and GP status; inclusion of these variables did not materially alter the estimates for the prespecified predictors. Among respondents expressing any preference, a secondary set of logistic regressions examined the direction of preference (female vs male physician) using the same predictor matrix.

***Operational modelling of preference fulfilment.*** For each scenario, the prevalence of any physician-gender preference and the proportion of preferrers who chose a female physician (p_0_) were estimated. Under random gender assignment, the expected fulfilment rate among preferrers is given by:

E[fulfilment] = p_0_ × f + (1 − p_0_) × (1 − f)

where f denotes the proportion of female physicians. This closed-form expectation was verified by Monte Carlo simulation (10,000 patients per run; fixed random seeds). A clinic session was modelled with a given female-physician share f and male-physician share (1 − f), assuming sufficient overall capacity such that matching depended only on gender mix. A preferrer requesting a female physician could be matched with probability f; one requesting a male physician with probability (1 − f). The primary outcome was the proportion of preferrers whose stated preference could be fulfilled. Extended regression outputs, directional-preference analyses, and simulation pseudocode are provided in Supplementary File 2.

### 2.5 Reporting standards

The study followed the STROBE (Strengthening the Reporting of Observational Studies in Epidemiology) guidelines for cross-sectional studies. A condensed STROBE checklist is provided in Supplementary File 2.

## 3 Results

### 3.1 Cohort and missingness

We analysed 324 respondents, whose demographic, clinical, and contextual characteristics are shown in Table 1. Two variables conditional on prior visits, last physician's gender and whether advice might have been better with a different physician gender, showed 36.73% missingness because first-time attendees were ineligible. This was treated as inapplicable rather than imputed. All other variables had 0.00 to 1.23% missingness and were imputed with the mode for categorical predictors before regression modelling to avoid unnecessary attrition.

**Table 1:** Demographic, clinical, and contextual characteristics of respondents (N = 324).

Variable	Category	n	% (of valid)
**Gender**	***Male***	***159***	***49.2 %***
	***Female***	***161***	***49.8 %***
	***Diverse***	***1***	***0.3 %***
	***Missing***	***3***	***0.9 %***
**Age group (years)**	***< 20***	***17***	***5.3 %***
	***20–39***	***62***	***19.2 %***
	***40–59***	***100***	***31.1 %***
	***60–79***	***113***	***35.2 %***
	***≥ 80***	***28***	***8.7 %***
	***Missing***	***4***	***1.2 %***
**Disease category**	***Tumour***	***115***	***35.5 %***
	***Spine (non-tumour)***	***148***	***45.7 %***
	***Other***	***59***	***18.2 %***
	***Missing***	***2***	***0.6 %***
**Education**	***Primary school (Grundschule)***	***26***	***8.0 %***
	***Lower secondary school (Hauptschule/Mittlere Reife)***	***125***	***38.6 %***
	***Upper secondary qualification (Abitur — equivalent to A-levels or high-school diploma)***	***93***	***28.7 %***
	***University degree (Bachelor or higher)***	***68***	***21.0 %***
	***No response***	***12***	***3.7 %***
**Religion**	***Protestant (Evangelical Church)***	***65***	***20.1 %***
	***Roman Catholic***	***26***	***8.0 %***
	***Other Christian denomination***	***17***	***5.2 %***
	***Muslim***	***13***	***4.0 %***
	***Jewish***	***5***	***1.5 %***
	***Other religion***	***12***	***3.7 %***
	***None (agnostic/atheist)***	***186***	***57.4 %***
**Immigration background**	***Yes (patient or at least one parent born abroad)***	***26***	***8.0 %***
	***No***	***279***	***86.1 %***
	***No response***	***19***	***5.9 %***
**First clinic visit**	***Yes***	***119***	***36.7 %***
	***No (return visit)***	***205***	***63.3 %***
**Ever requested specific-gender physician**	***Yes***	***34***	***10.5 %***
	***No***	***290***	***89.5 %***
**Symptom limits daily activities**	***Yes***	***187***	***57.7 %***
	***No***	***137***	***42.3 %***
**Symptom is worrying**	***Yes***	***168***	***51.9 %***
	***No***	***156***	***48.1 %***
**Symptom is painful**	***Yes***	***184***	***56.8 %***
	***No***	***140***	***43.2 %***
**Symptom is embarrassing**	***Yes***	***44***	***13.6 %***
	***No***	***280***	***86.4 %***

A post-hoc sensitivity analysis indicated that, given the sample size of 324 and an approximate baseline preference rate of 0.4, the study had 80% power to detect odds ratios of about 1.8 or greater. This suggests that smaller associations may not have reached statistical significance owing to limited precision.

### 3.2 Prevalence and direction of preferences

**Fig. 1:**
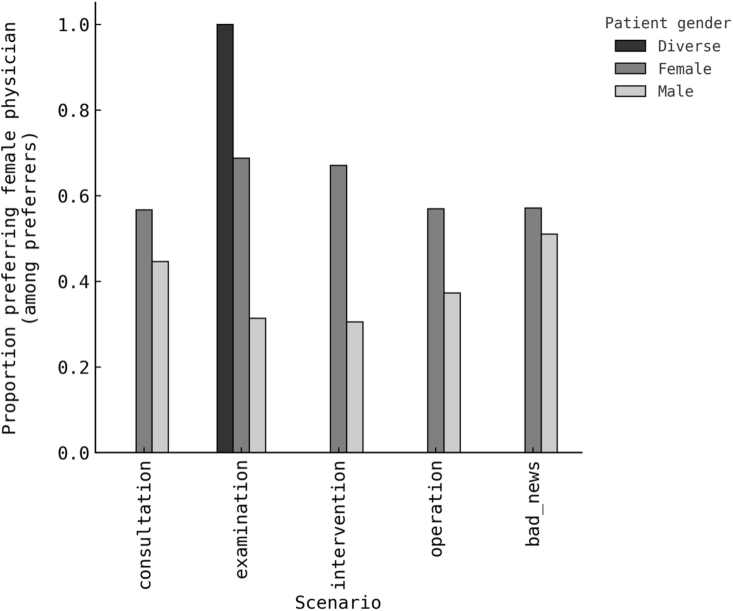
Prevalence of physician-gender preference by clinical scenario and patient gender (N = 324). Proportion of respondents reporting any gender preference (male or female) across five common neurosurgical outpatient contexts: consultation, consultation with physical examination, minor intervention under local anaesthesia, major operation under general anaesthesia, and disclosure of bad news. Bars represent proportions within each patient-gender group. “Diverse” denotes respondents outside the binary categories (n = 1); percentages are shown for completeness only, and this category was excluded from inferential analyses.

**Fig. 2:**
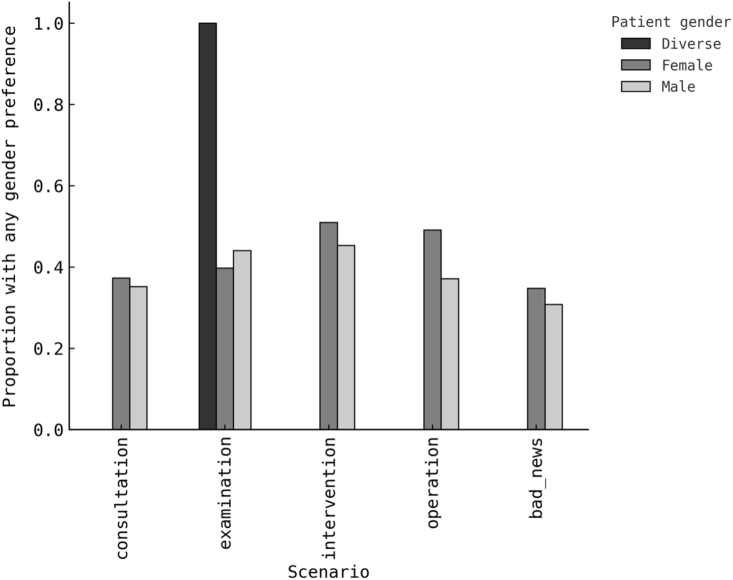
Direction of physician-gender preference among preferrers by clinical scenario and patient gender (N = 324). Proportion of respondents expressing a preference for a female physician among those who indicated any gender preference. Bars show distributions by patient gender. “Diverse” refers to respondents identifying outside the binary categories and is shown for completeness but not used for inferential analysis.

Any physician-gender preference was present in 35.8% for consultation, 42.3% for examination, 48.1% for minor intervention, 42.9% for operation, and 32.7% for disclosure of bad news. Among respondents with any preference, direction toward a female physician was balanced: 50.9% at consultation, 49.6% at examination, 50.0% at intervention, 48.2% at operation, and 53.8% at disclosure of bad news. These core estimates with numerators and denominators appear in Table 2 and are visualised in Fig. 1 for prevalence and Fig. 2 for direction among preferrers. The overall pattern is consistent with a largely preference-neutral population, with a higher prevalence of preference in scenarios that plausibly involve physical contact or discomfort.

**Table 2:** Physician-gender preference prevalence and direction by clinical scenario (N = 324).

Scenario	Respondents (n)	Any preference n	Any preference %	Female preference n/Preferrers n	Female direction % among preferrers
Consultation	324	116	35.8 %	59/116	50.9 %
Examination	324	137	42.3 %	68/137	49.6 %
Minor intervention (local anaesthesia)	324	156	48.1 %	78/156	50.0 %
Major operation (general anaesthesia)	324	139	42.9 %	67/139	48.2 %
Disclosure of bad news	324	106	32.7 %	57/106	53.8 %

The table summarises the proportion of respondents reporting a physician-gender preference across five typical neurosurgical outpatient contexts. “Any preference” denotes selection of either a male or female physician rather than “no preference.” Among respondents expressing a preference, the directional percentage indicates the proportion favouring a female physician. Percentages are based on valid responses for each scenario.

### 3.3 Determinants of any physician-gender preference

We fitted separate multivariable logistic models for each scenario with HC3-robust standard errors. Predictors were patient gender, age group, disease category, indicators of symptom burden (daily activity limitation, worry, pain, embarrassment), first-visit status, prior request for a specific physician gender, and immigration background. Coefficients are reported as odds ratios with 95% robust confidence intervals (Table 3).

**Table 3:** Determinants of any physician-gender preference (multivariable logistic regression).

Predictor	Consultation OR (95 % CI)	Examination OR (95 % CI)	Intervention OR (95 % CI)	Operation OR (95 % CI)	Bad-news OR (95 % CI)
**Patient gender (Male vs Female)**	**0.82 (0.51–1.33)**	**1.32 (0.82–2.12)**	**0.93 (0.58–1.47)**	**0.55 (0.34–0.89) ∗**	**0.91 (0.54–1.55)**
**Age 80+ vs 20**–**39**	**0.76 (0.30–1.94)**	**0.78 (0.31–1.97)**	**0.37 (0.14–0.98) ∗**	**0.71 (0.29–1.77)**	**0.94 (0.35–2.52)**
**Disease = Tumour (ref Other)**	**0.93 (0.47–1.83)**	**0.67 (0.34–1.32)**	**0.84 (0.43–1.66)**	**0.74 (0.38–1.44)**	**1.01 (0.49–2.09)**
**Symptom limits daily activities**	**1.28 (0.78–2.08)**	**1.53 (0.92–2.52)**	**1.39 (0.84–2.31)**	**1.31 (0.79–2.18)**	**1.11 (0.63–1.95)**
**Symptom is worrying**	**1.09 (0.69–1.73)**	**0.97 (0.60–1.57)**	**1.65 (1.03–2.66) ∗**	**0.58 (0.36–0.94) ∗**	**1.08 (0.66–1.76)**
**Pain present**	**1.08 (0.68–1.73)**	**0.81 (0.49–1.35)**	**1.12 (0.69–1.82)**	**1.03 (0.63–1.69)**	**0.90 (0.54–1.51)**
**Embarrassment present**	**0.79 (0.36–1.72)**	**0.91 (0.46–1.82)**	**1.09 (0.53–2.25)**	**0.91 (0.42–1.96)**	**1.34 (0.60–3.00)**
**First visit (vs return)**	**1.01 (0.63–1.61)**	**1.14 (0.71–1.86)**	**0.95 (0.59–1.54)**	**1.10 (0.67–1.80)**	**1.05 (0.63–1.74)**
**Prior specific-gender request**	**1.42 (0.68–2.97)**	**1.88 (0.88–3.99)**	**1.51 (0.73–3.13)**	**1.45 (0.68–3.10)**	**1.32 (0.61–2.88)**
**Immigration background**	**1.19 (0.63–2.26)**	**1.15 (0.59–2.24)**	**1.11 (0.58–2.12)**	**1.04 (0.55–1.97)**	**0.96 (0.49–1.90)**

Values are odds ratios (OR) with 95 % robust confidence intervals. Asterisks indicate p < 0.05. All predictors listed were included in each model. Variables relating to education, religion, and relationship status were not entered because preliminary univariable screening and collinearity diagnostics showed no significant associations with the outcome and minimal contribution to model fit. The final model therefore retained variables representing demographic characteristics (patient gender, age group, immigration background), clinical context (disease category, symptom burden, first-visit status), and prior behaviour (previous specific-gender request).

Across the five scenarios, most predictors showed modest, non-significant associations with the likelihood of expressing a physician-gender preference, indicating that preference expression was largely independent of basic demographic or clinical features. In the consultation and examination scenarios, no variable reached statistical significance, though small trends suggested fewer preferences among older patients. In the intervention scenario (minor procedure under local anaesthesia), worry about the presenting symptom was significantly associated with higher odds of expressing a gender preference (OR 1.65, 95% CI 1.03–2.66, p = 0.039), while patients aged ≥80 years were less likely to report any preference (OR 0.37, 95% CI 0.14–0.98, p = 0.046). For operation scenarios (major surgery under general anaesthesia), male gender was linked to a lower likelihood of preference (OR 0.55, 95% CI 0.34–0.89, p = 0.014), and worry again predicted reduced preference expression (OR 0.58, 95% CI 0.36–0.94, p = 0.027). In the bad-news scenario, no covariate achieved significance. Overall, the results indicate that emotional context—particularly symptom-related worry—and gender interact with the procedural nature of the scenario: anxiety amplified preferences for minor procedures, whereas in high-stakes or operative settings, it appeared to suppress them. No consistent associations were observed for pain, embarrassment, first-visit status, immigration background, or prior specific-gender request after adjustment.

**Fig. 3:**
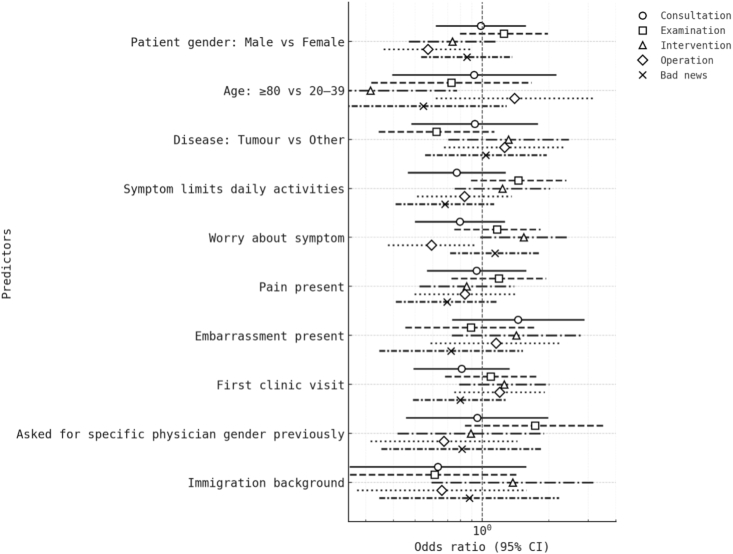
**Determinants of any physician-gender preference across neurosurgical scenarios.** Multivariable logistic regression models were fitted separately for each scenario. Points represent adjusted odds ratios with 95 % confidence intervals on a logarithmic scale. Distinct markers and line styles denote scenarios: ● Consultation, □ Examination, △ Minor intervention, ◇ Operation, and × Disclosure of bad news. Predictors included patient gender, age group, disease category, symptom burden (activity limitation, worry, pain, embarrassment), first-visit status, prior gender request, and immigration background. Most variables showed no significant association with preference expression. Significant effects were observed for symptom-related worry—increasing preference likelihood for minor interventions (OR 1.65, 95 % CI 1.03–2.66) but decreasing it for operations (OR 0.58, 95 % CI 0.36–0.94)—and for male gender in the operation scenario (OR 0.55, 95 % CI 0.34–0.89). The overall pattern suggests that emotional context, rather than demographic factors, primarily modulates the expression of physician-gender preference.

Fig. 3 summarises adjusted odds ratios for each predictor across all clinical scenarios. As shown, few variables exerted large effects, with significant associations limited to symptom-related worry (intervention and operation contexts) and patient gender (operation context).

### 3.4 Preference fulfilment under varying staffing compositions

Having established that preferences are common but balanced in direction, we next examined whether altering the workforce gender ratio could meaningfully improve preference fulfilment using the Monte Carlo simulation framework described in Methods.

**Fig. 4:**
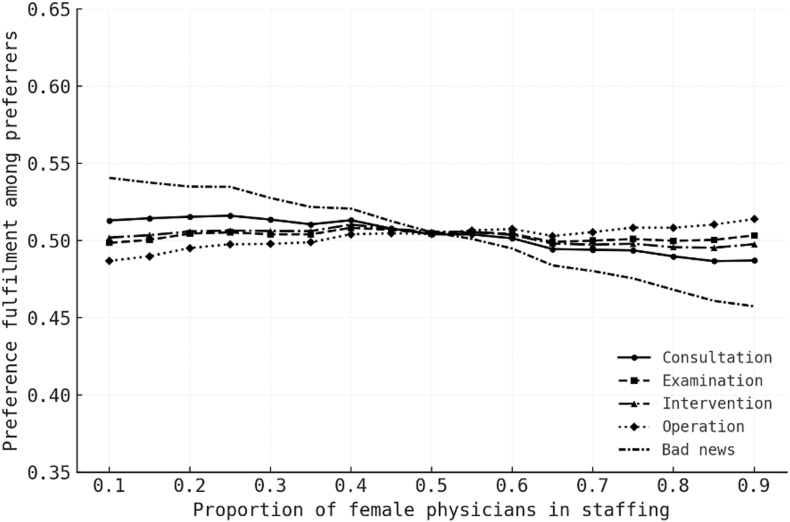
Operational feasibility of honouring preferences across staffing mixes. Plot depicts, for each scenario, the expected share of preferrers whose stated gender preference can be fulfilled as the female-physician share varies from 0.1 to 0.9. Distinct line styles and markers distinguish scenarios. Curves are nearly flat around 0.5 across all scenarios, with minor slope where directional preferences are imbalanced.

Across all scenarios, the expected fulfilment proportion for preferrers clustered near 50% for staffing compositions between 0.20 and 0.60 female physicians. This pattern reflects the near-symmetric directional preferences observed in Table 2. Small deviations from 50% occurred where directional preference was slightly imbalanced. For example, in the disclosure-of-bad-news scenario, where 53.8% of preferrers chose a female physician, fulfilment exceeded 50% when the female staff share was low and fell below 50% when it was high. Results for representative staffing mixes are shown in Table 4, and continuous curves across the full range of f in Fig. 4.

**Table 4:** Expected fulfilment of stated physician-gender preferences among preferrers by staffing mix.

Scenario	Female staff share = 0.2	0.3	0.4	0.5	0.6
**Consultation**	**50.7**	**50.4**	**50.3**	**49.6**	**50.1**
**Examination**	**50.0**	**50.0**	**49.9**	**49.8**	**50.3**
**Intervention**	**49.8**	**50.1**	**50.0**	**49.8**	**50.5**
**Operation**	**49.3**	**49.5**	**49.6**	**49.8**	**50.7**
**Bad news**	**52.7**	**51.7**	**51.2**	**49.8**	**49.6**

Proportion of preferrers whose stated preference can be honoured, simulated with 10,000 patients per run and fixed seeds. Entries are percentages. The small departures from 50 % are driven by slight imbalances in directional preference and their interaction with staffing mix. Simulated values match the closed-form expectation given in the text.

In summary, modifying the workforce gender ratio alone did not meaningfully improve the fulfilment of patient gender preferences in this cohort.

## 4 Discussion

### 4.1 Balanced preferences driven by emotional context

Approximately one-third to one-half of neurosurgical outpatients expressed a physician-gender preference, with the highest rates in scenarios involving physical contact or procedural discomfort. Crucially, direction among preferrers was balanced near 50% across all scenarios. After multivariable adjustment, the only consistent predictors were symptom-related worry and patient gender in the operative setting. An odds ratio of 1.65 for worry in the minor intervention scenario corresponds to an absolute increase of roughly 13 percentage points at the observed baseline, underscoring that even statistically significant effects were modest in practical terms. Simulation modelling confirmed that variations in the staff gender mix alone had negligible effect on preference fulfilment given these balanced directional tendencies.

### 4.2 Gender preferences across clinical specialties

The current results extend previous work on physician-gender preferences beyond primary care and gynaecology into a procedural, high-technology field. Prior studies have typically reported that 10–30% of patients hold explicit gender preferences, often motivated by perceived empathy, communication style, or comfort during examination (Dagostini et al., 2022; Fink et al., 2020; Lee et al., 2024). The higher prevalence observed in the present neurosurgical cohort suggests that contexts involving psychological vulnerability, decisional stress, or anticipated bodily exposure may heighten gender-related comfort considerations (Roter and Hall, 2004). Unlike in obstetric and urologic care, where clear same-gender preferences have been documented (Amir et al., 2016; Tam et al., 2020), no dominant directional bias emerged in neurosurgery—consistent with interactions that are typically less physically intimate and more cognitively or emotionally demanding.

The minimal influence of demographic covariates contrasts with reports in which younger, female, or more religious patients were more likely to express preferences. In our cohort, neither education, religion, nor immigration background contributed significantly after adjustment, possibly reflecting the regional sociocultural homogeneity of the Thuringian catchment area or the high baseline trust in university-affiliated care. The association between symptom-related worry and preference expression in minor procedures aligns with affective decision-making models, wherein anxiety heightens sensitivity to relational factors. In contrast, the inverse association in major operative settings suggests that once anxiety surpasses a certain threshold, technical competence and institutional reassurance may dominate relational considerations.

Beyond patient perceptions, emerging evidence suggests that surgeon gender may independently associate with clinical outcomes. In a population-based Swedish cohort of over 150,000 cholecystectomies, patients operated on by male surgeons had higher rates of surgical complications (OR 1.29, 95% CI 1.19–1.40) and bile duct injuries in elective cases (OR 1.69, 95% CI 1.22–2.34), while female surgeons operated more slowly (Blohm et al., 2023). At the team level, Hallet et al. (2024) found that hospitals with greater anaesthesia–surgery team sex diversity (>35% female) had lower odds of 90-day major morbidity (OR 0.97, 95% CI 0.95–0.99), with stronger effects among patients treated by female surgeons (OR 0.83, 95% CI 0.76–0.90). These surgical findings complement population-level medical data showing lower mortality among patients treated by female physicians (Tsugawa et al., 2017; Greenwood et al., 2018; Heybati et al., 2025), though the mechanisms likely involve communication patterns, clinical decision-making, and team dynamics rather than gender per se. In our neurosurgical outpatient cohort, the balanced directional preferences and weak demographic associations suggest that patient comfort is more situational and relational than trait-based, but the outcome data provide additional context for why gender composition in surgical teams warrants ongoing attention.

### 4.3 Workforce composition versus preference-aware scheduling

The simulations reaffirm that staff-gender composition exerts negligible influence on preference fulfilment under the empirically observed, near-symmetric directional preferences. Greater operational benefit is more plausibly achieved through preference-aware scheduling methods that allocate declared preferrers to matching appointment slots or use flexible assignment rules within existing templates. Similar optimisation frameworks in outpatient and radiology scheduling have shown that integrating patient preferences and adaptive slot management can enhance efficiency without additional staffing demands (Cayirli and Veral, 2003; Ahmadi-Javid et al., 2017; Brandenburg et al., 2015; Cayirli et al., 2006). In neurosurgery, such targeted flexibility could be implemented at the booking stage where preference data are available, without restructuring the broader outpatient system.

### 4.4 Communication quality as the dominant determinant

The absence of strong gender effects may be interpreted as indirect evidence that professionalism and high-quality communication in the neurosurgical outpatient setting render physician gender less salient. Street et al. (2008) found that perceived personal similarity and patient-centred communication were stronger predictors of trust and satisfaction than demographic concordance, and Yaqoob et al. (2025) confirmed that empathy, responsiveness, and continuity of care were central to neurosurgical patient satisfaction. Our finding that situational worry—rather than gender per se—predicted preference expression supports the notion that relational comfort is dynamic and context-sensitive. The near-neutral directional preference further suggests that merely increasing female representation among neurosurgeons is unlikely, by itself, to improve comfort metrics, although workforce equity remains a valid institutional goal.

### 4.5 Limitations

This study has several limitations. First, it was conducted at a single academic neurosurgical centre in central Germany; although the regional catchment is demographically typical, findings may not generalise to metropolitan or culturally diverse populations. Second, the cross-sectional design captures stated preferences rather than observed behaviour. Patients might respond differently when confronted with real scheduling trade-offs or in acute inpatient settings. Because continuity of physician assignment is limited in our outpatient setting, individual negative or positive experiences with a specific physician could still have influenced stated gender preferences. Third, the sample size, though adequate for prevalence estimates, limits detection of small effect sizes in multivariable models. Fourth, all scenarios were hypothetical; actual emotional salience during real clinical encounters could modify reported comfort. Fifth, while the synthetic anonymity of responses minimised social desirability bias, it also precluded linkage with objective clinical or satisfaction outcomes. Sixth, because the number of patients approached was not recorded, we cannot compute a response rate; hence the analysis reports only the evaluated cohort and item-level missingness, consistent with STROBE guidance (Vandenbroucke et al., 2014). Seventh, the Monte Carlo model simplified gender matching to binary categories and static staffing ratios; in practice, appointment clustering, subspecialty constraints, and non-binary identities would add complexity. Lastly, the “diverse” gender category was very small, precluding inferential modelling for non-binary identities. We also modelled binary physician gender; accommodation for non-binary and gender-diverse identities requires different matching rules and sufficient sample sizes.

### 4.6 Future directions

Future work should triangulate attitudinal and behavioural data by linking preference declarations to actual scheduling and satisfaction outcomes. Qualitative inquiry could further elucidate the psychological and communicative mechanisms underlying gender-related comfort in neurosurgical care, particularly during sensitive discussions such as prognostic disclosure or functional loss. From an operational standpoint, preference-aware scheduling models could be implemented prospectively to test whether targeted matching affects patient satisfaction, anxiety, or adherence to follow-up. Comparative analyses across surgical specialties may also clarify whether the neutral directional balance observed here represents a neurosurgery-specific phenomenon or a broader trend within procedural disciplines.

## 5 Conclusions

Gender preferences among neurosurgical outpatients were common but balanced and only weakly influenced by demographic or clinical characteristics. Emotional state, rather than gender identity itself, determined whether such preferences were expressed. Preferences were most likely to arise during examinations and minor interventions, where physical proximity and perceived vulnerability are greatest. Adjusting the workforce gender ratio alone yields negligible improvement. Where feasible, targeted preference-aware scheduling may modestly improve comfort in selected outpatient scenarios; however, its practical impact is limited. More consistently, awareness of situational anxiety and high-quality, empathic communication appear to outweigh physician gender itself.

## Ethical approval

The study was approved by the institutional ethics committee of Friedrich Schiller University Jena (reference 2023-2910-Bef). The ethics committee waived the requirement for written informed consent. The study adhered to the principles of the Declaration of Helsinki and the STROBE reporting guideline.

## Authorship statement

All authors meet ICMJE authorship criteria, contributed substantially to the conception, design, data collection, analysis, and interpretation, revised the manuscript critically for intellectual content, approved the final version, and agree to be accountable for all aspects of the work.

## Funding

This study received no dedicated external funding.

## Declaration of competing interest

The authors declare that they have no known competing financial interests or personal relationships that could have appeared to influence the work reported in this paper.

## Data Availability

The patient-level data underlying this study contain potentially re-identifiable information and therefore cannot be shared publicly in accordance with institutional and data protection regulations. Summary tabulations and analysis code are available from the corresponding author on reasonable request.
