# Supplementary material for: Patient gender preferences in neurosurgical care: A cross-sectional study with operational modelling
Source: Brain Spine. 2026 Apr 9;6:106035. doi: 10.1016/j.bas.2026.106035 (PMC13092189; doi:10.1016/j.bas.2026.106035)
Supplement: Multimedia component 1 [file mmc1.docx]

# Supplementary File 1

# Questionnaire

This is a reproduction of the questionnaire used in the 2023 study on patient gender preferences in the neurosurgical outpatient clinic. The English version below provides a faithful translation of the original German instrument.

## 1. Your gender

- Male
- Female
- Diverse / Other

## 2. Your age

- Under 20 years
- 20–39 years
- 40–59 years
- 60–79 years
- 80 years or older

## 3. Which of the following best describes your condition?

- Tumor disease
- Spinal disease, non-tumorous
- Other

## 4. The health problem for which I am here today…

- limits my daily activities
- is worrying
- is painful
- is embarrassing for me

## 5. Is today your first appointment in our department?

- Yes – if yes, continue with question 8
- No

## 6. If you have been here before, were you treated last time by a male or a female doctor?

- Male doctor
- Female doctor
- I don’t remember

## 7. Do you think the consultation at your last visit would have been better if it had been conducted by a doctor of the opposite gender?

- Yes
- No

## 8. Do you currently have a general practitioner (family doctor)?

- Male GP
- Female GP
- I do not currently have a GP

## 9. Have you ever requested to be treated by a doctor of a specific gender?

- Yes
- No

## 10. In the following situations, would you have a preference for the doctor’s gender? For each, choose: Male / Female / No preference

- Consultation only, without physical examination
- Consultation with physical examination
- Minor procedure under local anaesthesia (e.g., pain injection)
- Major operation under general anaesthesia

## 11. If a doctor had to deliver very bad news...

Male / Female / No preference

## 12. If you prefer to be treated by doctors of a particular gender, what are your reasons?

- Personal comfort and trust
- Previous experiences with doctors of this gender
- Belief that the doctor’s gender aligns with your personal values
- Perception of better communication and understanding
- Perception of better bedside manner
- Perception of greater empathy or compassion
- Belief that doctors of this gender are more knowledgeable or qualified
- Belief that doctors of this gender are more sensitive to specific health needs
- Other (please specify)
- I would not prefer one gender over another

## 13. What is your marital or relationship status?

- Single
- Married
- Divorced
- Widowed
- Civil partnership
- Prefer not to say

## 14. Were you or at least one of your parents born abroad?

- Yes
- No
- Prefer not to say

## 15. What is your highest level of education?

- Primary / elementary school
- Secondary school
- High school diploma
- University degree
- Prefer not to say

## 16. What is your religious affiliation?

- Protestant
- Roman Catholic
- Other Christian denomination
- Muslim
- Jewish
- Other
- None
