# Supplementary material for: Patient gender preferences in neurosurgical care: A cross-sectional study with operational modelling
Source: Brain Spine. 2026 Apr 9;6:106035. doi: 10.1016/j.bas.2026.106035 (PMC13092189; doi:10.1016/j.bas.2026.106035)
Supplement: Multimedia component 2 [file mmc2.docx]

# Supplementary File 2

# Extended Methods, Regression Outputs, and Simulation Details

## S1. Regression Model Specification

Multivariable logistic regression models were fitted separately for each scenario (consultation, examination, minor intervention, major operation, disclosure of bad news).

The general model form was:

$$logit(P\_i) = \beta₀ + \beta₁\cdot Male + \beta₂\cdot AgeGroup + \beta₃\cdot Tumour + \beta₄\cdot Spine + \beta₅\cdot ActivityLimit + \beta₆\cdot Worry + \beta₇\cdot Pain + \beta₈\cdot Embarrassment + \beta₉\cdot FirstVisit + \beta₁₀\cdot Immigration + \beta₁₁\cdot PriorRequest + \varepsilonᵢ$$

All predictors were entered simultaneously. Categorical variables were dummy-coded with fixed reference categories: female gender, age 20–39 years, and disease category “other”. Robust HC3 standard errors were applied. Analyses were conducted in Python 3.11 (statsmodels v0.14.0).

The disease category “other” served as the reference level in all models, and HC3-robust covariance matrices were used to account for mild heteroscedasticity.

## S2. Covariate Coding

| **Variable** | **Reference / Coding** |
| --- | --- |
| Gender (Male) | 1 = Male, 0 = Female (reference) |
| Age group | Categorical: <20, 20–39 (ref), 40–59, 60–79, ≥80 |
| Disease category | Categorical: tumour, spine, other (ref) |
| Symptom limits daily activities | 1 = Yes, 0 = No |
| Symptom is worrying | 1 = Yes, 0 = No |
| Symptom is painful | 1 = Yes, 0 = No |
| Symptom is embarrassing | 1 = Yes, 0 = No |
| First clinic visit | 1 = Yes, 0 = No |
| Immigration background | 1 = Yes, 0 = No |
| Prior specific-gender request | 1 = Yes, 0 = No |

**Supplementary Table S1. Covariate coding and reference categories**

*Independent variables entered into all multivariable logistic regression models. “Other” served as the reference category for disease type, and female gender as the reference for patient gender. All variables were binary or dummy-coded categorical predictors. These codes apply identically across all scenarios.*

## S3. Regression Outputs Summary

Full numerical regression outputs are omitted from publication due to institutional data protection regulations. The following results summarise key patterns across models:

- Significant associations (p < 0.05) were observed for symptom-related worry, which increased preference likelihood during minor interventions and decreased it for major operations.
- Male patient gender was associated with lower likelihood of expressing a preference in the operation scenario.
- Age ≥ 80 years predicted lower preference expression in the minor intervention scenario.
- No other covariate reached statistical significance (all p > 0.05); effect directions for pain, embarrassment, immigration background, first-visit status, or prior specific-gender request were small and inconsistent across scenarios.

| **Scenario** | **N** | **Log-Likelihood** | **McFadden R²** | **LR χ² (df)** | **p-value** |
| --- | --- | --- | --- | --- | --- |
| Consultation | 324 | −207.19 | 0.020 | 8.29 (14) | 0.874 |
| Examination | 324 | −215.74 | 0.022 | 9.93 (14) | 0.767 |
| Minor intervention | 324 | −215.74 | 0.038 | 17.23 (14) | 0.244 |
| Operation | 324 | −212.09 | 0.042 | 18.42 (14) | 0.188 |
| Disclosure of bad news | 324 | −200.33 | 0.022 | 8.98 (14) | 0.833 |

**Supplementary Table S2. Model fit statistics for multivariable logistic regressions of any physician-gender preference**

*Values derived from the prespecified multivariable logistic regression models listed in Table 3 of the main manuscript. McFadden R² is defined as 1 − (L_model / L_null). Likelihood-ratio (LR) χ² tests compare each model against an intercept-only model. Model fit was modest (McFadden R² ≈ 0.02 – 0.04), typical for attitudinal outcomes.*

## S4. Directional-Preference Models

Among respondents expressing any physician-gender preference, we modelled direction (female vs male physician) using logistic regression. No predictors achieved statistical significance after adjustment. Minor, non-significant trends suggested slightly greater female-physician preference among younger women and those citing embarrassment. Given balanced directional distributions (~50% each), these effects were not retained for operational modelling.

| **Predictor** | **Consultation OR (95 % CI)** | **Examination OR (95 % CI)** | **Minor Intervention OR (95 % CI)** | **Operation OR (95 % CI)** | **Bad News OR (95 % CI)** |
| --- | --- | --- | --- | --- | --- |
| Patient gender (Male) | 0.91 (0.54 – 1.56) | 0.97 (0.57 – 1.64) | 1.04 (0.61 – 1.77) | 0.88 (0.50 – 1.55) | 1.01 (0.55 – 1.87) |
| Age ≥ 80 y | 0.74 (0.27 – 2.03) | 0.68 (0.26 – 1.91) | 0.82 (0.30 – 2.23) | 0.93 (0.33 – 2.64) | 0.89 (0.31 – 2.57) |
| Symptom embarrassing | 1.41 (0.72 – 2.74) | 1.32 (0.69 – 2.51) | 1.46 (0.75 – 2.84) | 1.28 (0.64 – 2.55) | 1.38 (0.68 – 2.78) |
| Symptom worrying | 1.08 (0.64 – 1.82) | 1.15 (0.67 – 1.96) | 1.21 (0.70 – 2.09) | 0.95 (0.54 – 1.68) | 1.09 (0.60 – 1.98) |

**Supplementary Table S3. Directional-preference logistic-regression results (female vs male physician among preferrers)**

*Secondary multivariable logistic regressions restricted to respondents expressing any physician-gender preference. Dependent variable = direction of preference (female = 1, male = 0). No predictors achieved statistical significance after adjustment. N ≈ 140 preferrers per scenario. Effects are presented as odds ratios (OR) with 95 % robust confidence intervals.*

## S5. Monte Carlo Simulation Methods

Monte Carlo simulations assessed the operational feasibility of fulfilling expressed preferences under varying staff gender compositions.

For each scenario, the proportion of preferrers and directional split (female vs male) were used to generate simulated patient cohorts (n = 10 000 per run). Each preferrer was randomly matched to available physician gender according to a specified female-staff share *f* (0.20–0.60, in 0.10 increments). Fulfilment probability was computed as the proportion of preferrers whose preferred gender was available. Simulations used a fixed random seed (42) and identical sample size (n = 10 000) per scenario × staffing-fraction combination to ensure reproducibility.

The simulation assumed random patient–physician assignment within gender strata, without incorporating subspecialty focus, procedural expertise, or fixed clinic rosters. In actual departmental practice, subspecialty gender composition is heterogeneous: the vascular service is led by a female neurosurgeon, neuro-oncology and spine clinics are predominantly male-staffed, and the functional/pain service has a mixed-gender team. These structural differences inherently limit the extent to which gender matching can be achieved in daily operations. The model therefore reflects an idealised operational environment intended to isolate the theoretical influence of workforce gender composition rather than replicate the full complexity of institutional scheduling.

Pseudocode:

## for f in [0.20, 0.30, 0.40, 0.50, 0.60]:

## fulfilled = 0

## random.seed(42)

## for i in range(10000):

## if preferrer:

## if prefers_female and random() < f: fulfilled += 1

## elif prefers_male and random() > f: fulfilled += 1

## rate = fulfilled / total_preferrers

## S6. Simulation Results Summary

Across all scenarios, expected fulfilment of expressed preferences remained approximately 50 % for staffing compositions between 20 % and 60 % female physicians. Small deviations reflected minor imbalances in directional preference. Adjusting workforce gender composition alone therefore yields negligible gains; preference-aware scheduling (e.g., routing preferrers to matching slots) is operationally more efficient.

## S7. Data Protection and Reproducibility Statement

The analyses were performed on anonymised survey data collected without direct personal identifiers (no names, dates of birth, patient identifiers, or contact details were recorded). However, because the dataset contains detailed categorical variables (for example, age band, education level, religion, and disease category), indirect re-identification from unique variable combinations cannot be excluded. Consequently, individual-level data are not publicly available. Summary tables and the analysis code are available from the corresponding author on reasonable request and subject to institutional data-sharing approval.

## S8. Reporting Guideline Compliance

This study conforms to the STROBE (Strengthening the Reporting of Observational Studies in Epidemiology) guidelines for cross-sectional studies. This checklist summarises where each recommended item is addressed in the manuscript or supplementary materials.

| **Item No.** | **Recommendation** | **Location in Manuscript** |
| --- | --- | --- |
| 1 | Indicate the study’s design in the title or abstract; provide informative summary | Title, Abstract |
| 2–3 | Explain background, rationale, and specific objectives | Introduction |
| 4–5 | Describe study design, setting, locations, and data collection period | Methods – Study setting |
| 6 | Eligibility criteria and participant selection | Methods – Participants and survey instrument |
| 7–8 | Define outcomes, exposures, predictors, confounders; give sources of data | Methods – Outcomes, Survey instrument |
| 9 | Describe efforts to address potential sources of bias | Methods – anonymity, validation |
| 10 | Explain how study size was determined | Methods – Statistical analysis |
| 11 | Explain handling of quantitative variables and groupings | Methods – Statistical analysis |
| 12 | Describe statistical methods, confounding control, subgroup analyses, missing data handling, sensitivity analyses | Methods – Statistical analysis; Supplementary File 2 S1–S6 |
| 13–14 | Report participant numbers and characteristics | Results – Cohort and missingness; Table 1 |
| 15–16 | Provide outcome events and unadjusted/adjusted estimates with precision (95 % CI) | Results – Tables 2–4; Supplementary File 2 S3 |
| 17 | Report subgroup, interaction, and sensitivity analyses | Supplementary File 2 S4–S6 |
| 18–20 | Summarise results, discuss limitations and interpretation in context | Discussion |
| 21 | Discuss generalisability (external validity) | Limitations section |
| 22 | State funding sources and roles of funders | Funding statement |
| 23 | Describe ethics approval and consent procedures | Ethical approval section |

**Supplementary Table S4. STROBE checklist for cross-sectional studies**
